# A convergent relaxation of the Douglas–Rachford algorithm

**DOI:** 10.1007/s10589-018-9989-y

**Published:** 2018-03-06

**Authors:** Nguyen Hieu Thao

**Affiliations:** 1 Delft Center for Systems and Control, Delft University of Technology, 2628CD Delft, The Netherlands; 2 Department of Mathematics, School of Education, Can Tho University, Can Tho, Vietnam

**Keywords:** Almost averagedness, Picard iteration, Alternating projection method, Douglas–Rachford method, RAAR algorithm, Krasnoselski–Mann relaxation, Metric subregularity, Transversality, Collection of sets, Primary 49J53, 65K10, Secondary 49K40, 49M05, 49M27, 65K05, 90C26

## Abstract

This paper proposes an algorithm for solving structured optimization problems, which covers both the backward–backward and the Douglas–Rachford algorithms as special cases, and analyzes its convergence. The set of fixed points of the corresponding operator is characterized in several cases. Convergence criteria of the algorithm in terms of general fixed point iterations are established. When applied to nonconvex feasibility including potentially inconsistent problems, we prove local linear convergence results under mild assumptions on regularity of individual sets and of the collection of sets. In this special case, we refine known linear convergence criteria for the Douglas–Rachford (DR) algorithm. As a consequence, for feasibility problem with one of the sets being affine, we establish criteria for linear and sublinear convergence of convex combinations of the alternating projection and the DR methods. These results seem to be new. We also demonstrate the seemingly improved numerical performance of this algorithm compared to the RAAR algorithm for both consistent and inconsistent sparse feasibility problems.

## Introduction

Convergence analysis has been one of the central and very active applications of variational analysis and mathematical optimization. Examples of recent contributions to the theory of the field that have initiated efficient programs of analysis are [1, 2, 7, 38]. It is the common recipe emphasized in these and many other works that there are two key ingredients required in order to derive convergence of a numerical method (1) regularity of individual functions or sets such as *convexity* and *averaging property*, and (2) regularity of collections of functions or sets at their critical points such as *transversality*, *Kurdyka-Łojasiewicz property* and *metric subregularity*. As a result, the question about convergence of a solving method can often be reduced to checking whether certain regularity properties of the problem data are satisfied. There have been a considerable number of papers studying these two ingredients of convergence analysis in order to establish sharper convergence criteria in various circumstances, especially those applicable to algorithms for solving nonconvex problems [5, 12, 13, 19, 26, 27, 31–33, 38, 42, 45].

This paper suggests an algorithm called $T_{\lambda }$, which covers both the backward-backward and the DR algorithms as special cases of choosing the parameter $\lambda \in [0,1]$, and analyzes its convergence. When applied to feasibility problem for two sets one of which is affine, $T_{\lambda }$ is a convex combination of the alternating projection and the DR methods. On the other hand, $T_{\lambda }$ can be viewed as a relaxation of the DR algorithm. Motivation for relaxing the DR algorithm comes from the lack of stability of this algorithm when applied to inconsistent problems. This phenomenon has been observed for the *Fourier phase retrieval problem* which is essentially inconsistent due to the reciprocal relationship between the spatial and frequency variables of the Fourier transform [35, 36]. To address this issue, a relaxation of the DR algorithm, often known as the RAAR algorithm, was proposed and applied to phase retrieval problems by Luke in the aforementioned papers. In the framework of feasibility, the RAAR algorithm is described as a convex combination of the basic DR operator and one of the projectors. Our preliminary numerical experiments have revealed a promising performance of algorithm $T_{\lambda }$ in comparison with the RAAR method. This observation has motivated the study of convergence analysis of algorithm $T_{\lambda }$ in this paper.

After introducing the notation and proving preliminary results in Sect. 2, we introduce $T_{\lambda }$ as a general fixed point operator, characterize the set of fixed points of $T_{\lambda }$ (Proposition 1), and establish abstract convergence criteria for iterations generated by $T_{\lambda }$ (Theorem 2) in Sect. 3. We discuss algorithm $T_{\lambda }$ in the framework of feasibility problems in Sect. 4. The set of fixed points of $T_{\lambda }$ is characterized for convex inconsistent feasibility (Proposition 3). For consistent feasibility we show that almost averagedness of $T_{\lambda }$ (Proposition 4) and metric subregularity of $T_{\lambda }-\,\mathrm{Id}$ (Lemma 3) can be obtained from regular properties of the individual sets and of the collection of sets, respectively. As a result, the two regularity notions are combined to yield local linear convergence of iterations generated by $T_{\lambda }$ (Theorem 4). Section 5 is devoted to demonstrate the improved numerical performance of algorithm $T_{\lambda }$ compared to the RAAR algorithm for both consistent and inconsistent feasibility problems. In this section, we study the feasibility approach for solving the *sparse optimization problem*. Our linear convergence result established in Sect. 4 for iterations generated by $T_{\lambda }$ is also illustrated in this application (Theorem 5).

## Notation and preliminary results

Our notation is standard, c.f. [11, 40, 46]. The setting throughout this paper is a finite dimensional Euclidean space ${\mathbb {E}}$. The norm $\Vert \cdot \Vert$ denotes the Euclidean norm. The open unit ball in a Euclidean space is denoted $\mathbb {B}$, and $\mathbb {B}_\delta (x)$ stands for the open ball with radius $\delta >0$ and center *x*. The distance to a set ${A}\subset {\mathbb {E}}$ with respect to the bivariate function $\text {dist}\,(\cdot , \cdot )$ is defined by

$$\begin{aligned} \text {dist}\,(\cdot , {A}) :{\mathbb {E}}\rightarrow {\mathbb {R}}_+:x\mapsto \inf _{y\in {A}}\text {dist}\,(x,y). \end{aligned}$$

We use the convention that the distance to the empty set is $+\infty$. The set-valued mapping

$$\begin{aligned} P_{A}:\,{\mathbb {E}}\rightrightarrows {\mathbb {E}}\,:x\mapsto \left\{ y\in {A}\,\left| \,\text {dist}\,(x,y)=\text {dist}\,(x,{A})\right. \right\} \end{aligned}$$

is the *projector* on *A*. An element $y\in P_{A}(x)$ is called a *projection*. This exists for any closed set ${A}\subset {\mathbb {E}}$. Note that the projector is not, in general, single-valued. Closely related to the projector is the *prox* mapping corresponding to a function *f* and a stepsize $\tau >0$ [41]

$$\begin{aligned} \text {prox}_{\tau ,f}(x):={\text {argmin}\,}_{y\in \mathbb {E}}\left\{ f(y)+\tfrac{1}{2\tau }\left\| y-x\right\| ^2\right\} . \end{aligned}$$

When $f=\iota _{A}$ is the *indicator function* of *A*, that is $\iota _{A}(x)=0$ if $x\in A$ and $\iota _{A}(x)=+\infty$ otherwise, then $\text {prox}_{\tau ,\iota _{A}}=P_{A}$ for all $\tau >0$. The *inverse of the projector*, $P^{-1}_{A}$, is defined by

$$\begin{aligned} P^{-1}_{A}(a):=\left\{ x\in {\mathbb {E}}\,\left| \,a\in P_{A}(x)\right. \right\} . \end{aligned}$$

The *proximal normal cone* to *A* at ${\bar{x}}$ is the set, which need not be either closed or convex,

1 $$\begin{aligned} N^{\mathrm{prox}}_{{A}}({\bar{x}}):=\text {cone}\,\left( P_{A}^{-1}({\bar{x}})-{\bar{x}}\right) . \end{aligned}$$

If ${\bar{x}}\notin {A}$, then $N^{\mathrm{prox}}_{{A}}({\bar{x}})$ is defined to be empty. Normal cones are central to characterizations both of the regularity of individual sets and of the regularity of collections of sets. For a refined numerical analysis of projection methods, one also defines the $\varLambda$-*proximal normal cone* to *A* at $\bar{x}$ by

$$\begin{aligned} N^{\mathrm{prox}}_{A|\varLambda }({\bar{x}}):=\text {cone}\,\left( \left( P_A^{-1}({\bar{x}})\cap \varLambda \right) -{\bar{x}}\right) . \end{aligned}$$

When $\varLambda ={\mathbb {E}}$, it coincides with the proximal normal cone (1).

For $\varepsilon \ge 0$ and $\delta >0$, a set *A* is $(\varepsilon ,\delta )$-*regular* relative to $\varLambda$ at $\bar{x}\in A$ [13, Definition 2.9] if for all $x\in {\mathbb {B}}_{\delta }(\bar{x})$, $a\in A\cap {\mathbb {B}}_{\delta }(\bar{x})$ and $v \in N^{\mathrm{prox}}_{A|\varLambda }(a)$,

$$\begin{aligned} \left\langle x-a, v \right\rangle \le \varepsilon \left\| x-a\right\| \left\| v\right\| . \end{aligned}$$

When $\varLambda =\mathbb {E}$, the quantifier “relative to” is dropped.

For a set-valued operator $T:{\mathbb {E}}\rightrightarrows {\mathbb {E}}$, its *fixed point set* is defined by ${{\mathrm{\mathsf {Fix}\,}}}T:=\left\{ x\in \mathbb {E}\,\left| \,x\in Tx\right. \right\}$. For a number $\lambda \in [0,1]$, we denote the $\lambda$-*reflector* of *T* by $R_{T,\lambda }:=(1+\lambda )T-\lambda \,\mathrm{Id}$. A frequently used example in this paper corresponds to *T* being a projector.

In the context of convergence analysis of Picard iterations, the following generalization of the Fejér monotonicity of sequences appears frequently, see, for example, the book [4] or the paper [39] for the terminology.

### Definition 1

(*Linear monotonicity*) The sequence $(x_k)$ is *linearly monotone* with respect to a set $S\subset {\mathbb {E}}$ with rate $c\in [0,1]$ if

$$\begin{aligned} \text {dist}\,(x_{k+1},S) \le c \,\text {dist}\,(x_k,S)\quad \forall k\in \mathbb {N}. \end{aligned}$$

Our analysis follows the abstract analysis program proposed in [38] which requires the two key components of the convergence: *almost averagedness* and *metric subregularity*.

### Definition 2

(*Almost nonexpansive/averaging mappings*) [38] Let $T:{\mathbb {E}} \rightrightarrows {\mathbb {E}}$ and $U\subset {\mathbb {E}}$.

(i) *T* is *pointwise almost nonexpansive* at *y* on *U* with violation $\varepsilon \ge 0$ if for all $x\in U$, $x^+\in Tx$ and $y^+\in Ty$,
  $$\begin{aligned} \left\| x^+- y^+\right\| \le \sqrt{1+\varepsilon }\left\| x-y\right\| . \end{aligned}$$
(ii) *T* is *pointwise almost averaging* at *y* on *U* with violation $\varepsilon \ge 0$ and averaging constant $\alpha >0$ if for all $x\in U$, $x^+\in Tx$ and $y^+\in Ty$,
  2 $$\begin{aligned} \left\| x^+- y^+\right\| ^2 \le \left( 1+\varepsilon \right) \left\| x-y\right\| ^2 - \frac{1-\alpha }{\alpha }\left\| (x^+-x)-(y^+-y)\right\| ^2. \end{aligned}$$

When a property holds at all $y\in U$ on *U*, we simply say that the property holds on *U*.

From Definition 2, almost nonexpansiveness is actually the almost averaging property with the same violation and averaging constant $\alpha =1$.

### Remark 1

(the range of quantitative constants) In the context of Definition 2, it is natural to consider *violation* $\varepsilon \ge 0$ and *averaging constant* $\alpha \in (0,1]$. Mathematically, it also makes sense to consider $\varepsilon <0$ and $\alpha >1$ provided that the required estimate (2) holds true. Simple examples for the later case are linear contraction mappings. In this paper, averaging constant $\alpha >1$ will frequently be involved implicitly in intermediate steps of our analysis without any contradiction or confusion. This is the reason why in Definition 2 (ii) we considered $\alpha >0$ instead of $\alpha \in (0,1]$ as in [38, Definition 2.2].

It is worth noting that if the iteration $x_{k+1}\in Tx_{k}$ is linearly monotone with respect to ${{\mathrm{\mathsf {Fix}\,}}}T$ with rate $c \in (0,1)$ and *T* is almost averaging on some neighborhood of ${{\mathrm{\mathsf {Fix}\,}}}T$ with averaging constant $\alpha \in (0,1]$, then $(x_k)$ converges R-linearly to a fixed point of *T* [39, Proposition 3.5].

We next prove a fundamental preliminary result for our analysis regarding almost averaging mappings.

### Lemma 1

Let $T:{\mathbb {E}}\rightrightarrows {\mathbb {E}}$, $U\subset {\mathbb {E}}$, $\lambda \in [0,1]$, $\varepsilon \ge 0$ and $\alpha >0$. The following two statements are equivalent.

(i) *T* is almost averaging on *U* with violation $\varepsilon$ and averaging constant $\alpha$.
(ii) The $\lambda$-reflector of *T*, $R_{T,\lambda }=(1+\lambda )T - \lambda \,\mathrm{Id}$, is almost averaging on *U* with violation $(1+\lambda )\varepsilon$ and averaging constant $(1+\lambda )\alpha$.

### Proof

Take any $x,y\in U$, $x^+\in Tx$, $y^+\in Ty$, $\tilde{x} = (1+\lambda )x^+-\lambda x \in R_{T,\lambda }x$ and $\tilde{y} = (1+\lambda )y^+-\lambda y \in R_{T,\lambda }y$. We have by definition of $R_{T,\lambda }$ and [4, Corollary 2.14] that

3 $$\begin{aligned} \left\| \tilde{x}-\tilde{y}\right\| ^2&= \left\| (1+\lambda )(x^+-y^+) -\lambda (x-y)\right\| ^2 \nonumber \\&=\; (1+\lambda )\left\| x^+-y^+\right\| ^2 - \lambda \left\| x-y\right\| ^2 + \lambda (1+\lambda )\left\| (x^+-x)-(y^+-y)\right\| ^2. \end{aligned}$$

We also note that

4 $$\begin{aligned} \left\| (\tilde{x}-x)-(\tilde{y}-y)\right\| = (1+\lambda )\left\| (x^+-x)-(y^+-y)\right\| . \end{aligned}$$

(i) $\Rightarrow$ (ii). Suppose that *T* is almost averaging on *U* with violation $\varepsilon$ and averaging constant $\alpha$. Substituting (2) into (3) and using (4), we obtain that

5 $$\begin{aligned}&\left\| \tilde{x}-\tilde{y}\right\| ^2 \nonumber \\&\quad \le \; (1+(1+\lambda )\varepsilon )\left\| x-y\right\| ^2 - (1+\lambda )\left( \frac{1-\alpha }{\alpha }-\lambda \right) \left\| (x^+-x)-(y^+-y)\right\| ^2 \nonumber \\&\quad =\; (1+(1+\lambda )\varepsilon )\left\| x-y\right\| ^2 - \frac{\frac{1-\alpha }{\alpha }-\lambda }{1+\lambda }\left\| (\tilde{x}- x)-(\tilde{y}-y)\right\| ^2 \nonumber \\&\quad =\; (1+(1+\lambda )\varepsilon )\left\| x-y\right\| ^2 - \frac{1-(1+\lambda )\alpha }{(1+\lambda )\alpha }\left\| (\tilde{x}- x)-(\tilde{y}-y)\right\| ^2, \end{aligned}$$

which means that $R_{T,\lambda }$ is almost averaging on *U* with violation $(1+\lambda )\varepsilon$ and averaging constant $(1+\lambda )\alpha$.

(ii) $\Rightarrow$ (i). Suppose that $R_{T,\lambda }$ is almost averaging on *U* with violation $(1+\lambda )\varepsilon$ and averaging constant $(1+\lambda )\alpha$, that is, the inequality (5) is satisfied. Substituting (3) into (5) and using (4), we obtain

$$\begin{aligned}&(1+\lambda )\left\| x^+-y^+\right\| ^2 - \lambda \left\| x-y\right\| ^2 + \lambda (1+\lambda )\left\| (x^+-x)-(y^+-y)\right\| ^2 \\&\quad \le \; (1+(1+\lambda )\varepsilon )\left\| x-y\right\| ^2 - \left( 1+\lambda \right) \left( \frac{1-\alpha }{\alpha }-\lambda \right) \left\| (x^+-x)-(y^+-y)\right\| ^2. \end{aligned}$$

Equivalently,

$$\begin{aligned} \left\| x^+-y^+\right\| ^2 \le (1+\varepsilon )\left\| x-y\right\| ^2 - \frac{1-\alpha }{\alpha }\left\| (x^+-x)-(y^+-y)\right\| ^2. \end{aligned}$$

Hence *T* is almost averaging on *U* with violation $\varepsilon$ and averaging constant $\alpha$ and the proof is complete. $\square$

Lemma 1 generalizes [13, Lemma 2.4] where the result was proved for $\alpha =1/2$ and $\lambda =1$.

The next lemma recalls facts regarding the almost averagedness of projectors and reflectors associated with regular sets.

### Lemma 2

Let $A\subset {\mathbb {E}}$ be closed and $(\varepsilon ,\delta )$-regular at $\bar{x}\in A$ and define

$$\begin{aligned} U:=\{x\in {\mathbb {E}}\mid P_Ax\subset {\mathbb {B}}_{\delta }(\bar{x})\}. \end{aligned}$$

(i) The projector $P_A$ is pointwise almost nonexpansive on *U* at every point $z\in A\cap {\mathbb {B}}_{\delta }(\bar{x})$ with violation $2\varepsilon +\varepsilon ^2$.
(ii) The projector $P_A$ is pointwise almost averaging on *U* at every point $z\in A\cap {\mathbb {B}}_{\delta }(\bar{x})$ with violation $2\varepsilon +2\varepsilon ^2$ and averaging constant 1 / 2.
(iii) The $\lambda$-reflector $R_{P_A,\lambda }$ is pointwise almost averaging on *U* at every point $z\in A\cap {\mathbb {B}}_{\delta }(\bar{x})$ with violation $(1+\lambda )(2\varepsilon +2\varepsilon ^2)$ and averaging constant $\frac{1+\lambda }{2}$.

### Proof

Statements (i) and (ii) can be found in [13, Theorem 2.14] or [38, Theorem 3.1 (i) & (iii)]. Statement (iii) follows from (ii) and Lemma 1 applied to $T=P_A$ and $\alpha =1/2$. $\square$

The following concept of *metric subregularity with functional modulus* has played a central role, explicitly or implicitly, in the convergence analysis of Picard iterations [1, 13, 38, 39]. Recall that a function $\mu :[0,\infty ) \rightarrow [0,\infty )$ is a *gauge function* if $\mu$ is continuous and strictly increasing and $\mu (0)=0$.

### Definition 3

(*Metric subregularity with functional modulus*) A mapping $F:\,{\mathbb {E}}\rightrightarrows {\mathbb {E}}\,$ is *metrically subregular with gauge *$\mu$ *on* $U\subset {\mathbb {E}}$ *for* *y* *relative to* $\varLambda \subset {\mathbb {E}}$ if

$$\begin{aligned} \mu \left( \text {dist}\,\left( x,{F}^{-1}(y)\cap \varLambda \right) \right) \le \text {dist}\,\left( y,{F}(x)\right) \quad \forall x\in U\cap \varLambda . \end{aligned}$$

When $\mu$ is a linear function, that is $\mu (t)=\kappa t,\, \forall t\in [0,\infty )$, one says “with constant $\kappa$” instead of “with gauge $\mu =\kappa \,\mathrm{Id}$”. When $\varLambda ={\mathbb {E}}$, the quantifier “relative to” is dropped.

Metric subregularity has many important applications in variational analysis and mathematical optimization, see the monographs and papers [11, 15–18, 20, 21, 25, 40, 44]. For the discussion of metric subregularity in connection with subtransversality of collections of sets, we refer the reader to [23, 24, 29, 30].

The next theorem serves as the basic template for the quantitative convergence analysis of fixed point iterations. By the notation $T:\,\varLambda \rightrightarrows \varLambda \,$ where $\varLambda$ is a subset of ${\mathbb {E}}$, we mean that $T:\,{\mathbb {E}}\rightrightarrows {\mathbb {E}}\,$ and $Tx\subset \varLambda$ for all $x\in \varLambda$. This simplification of notation should not lead to any confusion if one keeps in mind that there may exist fixed points of *T* that are not in $\varLambda$. For the importance of the use of $\varLambda$ in isolating the desirable fixed point, we refer the reader to [1, Example 1.8]. In the following, ${\text {ri}}\, \varLambda$ denotes the *relative interior* of $\varLambda$.

### Theorem 1

[38, Theorem 2.1] Let $T:\,\varLambda \rightrightarrows \varLambda \,$ for $\varLambda \subset {\mathbb {E}}$ and let $S\subset {\text {ri}}\, \varLambda$ be closed and nonempty such that $Ty \subset {{\mathrm{\mathsf {Fix}\,}}}T \cap S$ for all $y\in S$. Let ${\mathcal {O}}$ be a neighborhood of *S* such that ${\mathcal {O}}\cap \varLambda \subset {\text {ri}}\, \varLambda$. Suppose that

- T is pointwise almost averaging at all points $y\in S$ with violation $\varepsilon$ and averaging constant $\alpha \in (0,1)$ on ${\mathcal {O}}\cap \varLambda$, and
- there exists a neighborhood ${\mathcal {V}}$ of ${{\mathrm{\mathsf {Fix}\,}}}T \cap S$ and a constant $\kappa >0$ such that for all $y\in S$, $y^+\in Ty$ and all $x^+\in Tx$ the estimate
  6 $$\begin{aligned} \kappa \,\text {dist}\,(x,S)\le \left\| \left( x-x^+\right) -\left( y-y^+\right) \right\| \end{aligned}$$
  holds whenever $$x\in \left( {\mathcal {O}}\cap \varLambda \right) \setminus \left( {\mathcal {V}}\cap \varLambda \right) $$.

Then for all $x^+\in Tx$

$$\begin{aligned} \text {dist}\,\left( x^+,{{\mathrm{\mathsf {Fix}\,}}}T\cap S\right) \le \sqrt{1+\varepsilon - \frac{(1-\alpha )\kappa ^2}{\alpha }}\,\text {dist}\,(x,S) \end{aligned}$$

whenever $$x\in \left( {\mathcal {O}}\cap \varLambda \right) \setminus \left( {\mathcal {V}}\cap \varLambda \right) $$.

In particular, if $\kappa >\sqrt{\frac{\varepsilon \alpha }{1-\alpha }}$, then for any initial point $x_0\in {\mathcal {O}}\cap \varLambda$ the iteration $x_{k+1}\in Tx_k$ satisfies

$$\begin{aligned} \text {dist}\,\left( x_{k+1},{{\mathrm{\mathsf {Fix}\,}}}T\cap S\right) \le c^k \,\text {dist}\,(x_0,S) \end{aligned}$$

with $c:=\sqrt{1+\varepsilon -\frac{(1-\alpha )\kappa ^2}{\alpha }}<1$ for all *k* such that $x_j\in \left( {\mathcal {O}}\cap \varLambda \right) \setminus \left( {\mathcal {V}}\cap \varLambda \right)$ for $j=1,2,\dots ,k$.

### Remark 2

[38, p. 13] In the case of $S={{\mathrm{\mathsf {Fix}\,}}}T$ condition (6) reduces to metric subregularity of the mapping ${F}:=T-\,\mathrm{Id}$ for 0 on the annular set $\left( {\mathcal {O}}\cap \varLambda \right) \setminus \left( {\mathcal {V}}\cap \varLambda \right)$, that is

$$\begin{aligned} \kappa \,\text {dist}\,(x,{F}^{-1}(0))\le \text {dist}\,(0,{F}(x))\quad \forall x\in \left( {\mathcal {O}}\cap \varLambda \right) \setminus \left( {\mathcal {V}}\cap \varLambda \right) . \end{aligned}$$

The inequality $\kappa >\sqrt{\frac{\varepsilon \alpha }{1-\alpha }}$ then states that the constant of metric subregularity $\kappa$ is sufficiently large relative to the violation of the averaging property of *T* to guarantee linear progression of the iterates through that annular region.

For a comprehensive discussion on the roles of *S* and $\varLambda$ in the analysis program of Theorem 1, we would like to refer the reader to the paper [38].

For the sake of simplification in terms of presentation, we have chosen to reduce the number of technical constants appearing in the analysis. It would be obviously analogous to formulate more theoretically general results by using more technical constants in appropriate places.

## $T_{\lambda }$ as a fixed point operator

We consider the problem of finding a fixed point of the operator

7 $$\begin{aligned} T_{\lambda } :=T_1\left( (1+\lambda )T_2 - \lambda \,\mathrm{Id}\right) - \lambda \left( T_2-\,\mathrm{Id}\right) , \end{aligned}$$

where $\lambda \in [0,1]$ and $T_i:{\mathbb {E}} \rightrightarrows {\mathbb {E}}$ $(i=1,2)$ are assumed to be easily computed.

Examples of $T_{\lambda }$ include the backward-backward and the DR algorithms [8, 10, 34, 36, 43] for solving the structured optimization problem

$$\begin{aligned} \underset{x\in {\mathbb {E}}}{\text{ minimize }}~f_1(x)+f_2(x) \end{aligned}$$

under different assumptions on the functions $f_i$ $(i=1,2)$. Indeed, when $T_i$ are the prox mappings of $f_i$ with parameters $\tau _i>0$, then $T_{\lambda }$ with $\lambda =0$ and 1 takes the form $T_{\lambda } = \text {prox}_{\tau _1,f_1}\circ \text {prox}_{\tau _2,f_2},$ and $T_{\lambda } = \text {prox}_{\tau _1,f_1}\left( 2\text {prox}_{\tau _2,f_2}-\,\mathrm{Id}\right) - \text {prox}_{\tau _2,f_2} +\,\mathrm{Id}$, respectively.

We first characterize the set of fixed points of $T_{\lambda }$ via those of the constituent operators $T_i$ $(i=1,2)$.

### Proposition 1

Let $T_1,T_2: {\mathbb {E}}\rightrightarrows {\mathbb {E}}$, $\lambda \in [0,1]$ and consider $T_{\lambda }$ defined at (7). The following statements hold true.

(i) $$(1+\lambda )T_{\lambda }-\lambda \,\mathrm{Id}=\left( (1+\lambda )T_1-\lambda \,\mathrm{Id}\right) \circ \left( (1+\lambda )T_2-\lambda \,\mathrm{Id}\right) $$.
  As a consequence,
  $$\begin{aligned} {{\mathrm{\mathsf {Fix}\,}}}T_{\lambda }={{\mathrm{\mathsf {Fix}\,}}}\left( (1+\lambda )T_1-\lambda \,\mathrm{Id}\right) \circ \left( (1+\lambda )T_2-\lambda \,\mathrm{Id}\right) . \end{aligned}$$
(ii) Suppose that $T_1=P_A$ is the projector on an affine set *A* and $T_2$ is single-valued. Then
  8 $$\begin{aligned} {{\mathrm{\mathsf {Fix}\,}}}T_{\lambda }&=\{x\in {\mathbb {E}} \mid P_Ax =\lambda T_2x+(1-\lambda )x\} \nonumber \\&\subset \{x\in {\mathbb {E}} \mid P_Ax = P_AT_2x\}. \end{aligned}$$

### Proof

(i). We have by the construction of $T_{\lambda }$ that

$$\begin{aligned} (1+\lambda )T_{\lambda }-\lambda \,\mathrm{Id}&=(1+\lambda )\left( T_1\left( (1+\lambda )T_2-\lambda \,\mathrm{Id}\right) -\lambda (T_2-\,\mathrm{Id})\right) -\lambda \,\mathrm{Id}\\&=(1+\lambda )T_1\left( (1+\lambda )T_2-\lambda \,\mathrm{Id}\right) - \lambda \left[ (1+\lambda )T_2-\lambda \,\mathrm{Id}\right] \\&=\left( (1+\lambda )T_1-\lambda \,\mathrm{Id}\right) \circ \left( (1+\lambda )T_2-\lambda \,\mathrm{Id}\right) . \end{aligned}$$

(ii). We first take an arbitrary $x\in {{\mathrm{\mathsf {Fix}\,}}}T_{\lambda }$ and prove that

$$\begin{aligned} P_Ax = P_AT_2x = \lambda T_2x + (1-\lambda )x. \end{aligned}$$

Indeed, from $x = T_{\lambda }x$, we get

9 $$\begin{aligned} x&= P_A\left( (1+\lambda )T_2x-\lambda x\right) -\lambda (T_2x-x) \nonumber \\&\Leftrightarrow \; \lambda T_2x+(1-\lambda )x=P_A\left( (1+\lambda )T_2x-\lambda x\right) . \end{aligned}$$

In particular, $\lambda T_2x+(1-\lambda )x\in A$. Thus by equality (9) and the assumption that $P_A$ is affine, we have

10 $$\begin{aligned} P_A\left( \lambda T_2x+(1-\lambda )x\right)&= P_A\left( (1+\lambda )T_2x-\lambda x\right) \nonumber \\ \Leftrightarrow \lambda P_AT_2x + (1-\lambda )P_Ax&= (1+\lambda )P_AT_2x-\lambda P_Ax \nonumber \\ \Leftrightarrow P_Ax&= P_AT_2x. \end{aligned}$$

Substituting (10) into (9) also yields

$$\begin{aligned} \lambda T_2x+(1-\lambda )x&= (1+\lambda )P_AT_2x-\lambda P_Ax \\&= (1+\lambda )P_Ax-\lambda P_Ax = P_Ax. \end{aligned}$$

Finally, let us take an arbitrary *x* satisfying $P_Ax = \lambda T_2x + (1-\lambda )x$ and prove that $x\in {{\mathrm{\mathsf {Fix}\,}}}T_{\lambda }$. Indeed, we note that $\lambda T_2x + (1-\lambda )x \in A$. Since $P_A$ is affine, one can easily check (10) and then (9), which is equivalent to $x\in {{\mathrm{\mathsf {Fix}\,}}}T_{\lambda }$. The proof is complete. $\square$

The inclusion (8) in Proposition 1 can be strict as shown in the next example.

### Example 1

Let us consider ${\mathbb {E}}={\mathbb {R}}^2$, the set $A=\left\{ (x_1,x_2)\in {\mathbb {R}}^2 \mid x_1=0\right\}$ and the two operators $T_1=P_A$ and $T_2x = \frac{1}{2}x$ $(\forall x\in {\mathbb {R}}^2)$. Then for any point $x=(x_1,0)$ with $x_1\ne 0$, we have $P_Ax = P_AT_2x=(0,0)$ but $P_Ax = (0,0) \ne (1-\lambda /2)x = \lambda T_2x + (1-\lambda )x$, that is $x\notin {{\mathrm{\mathsf {Fix}\,}}}T_{\lambda }$.

The next proposition shows that the almost averagedness of $T_{\lambda }$ naturally inherits from that of $T_1$ and $T_2$ via Krasnoselski–Mann relaxations.

### Proposition 2

(Almost averagedness of $T_{\lambda }$) Let $\lambda \in [0,1]$, $T_i$ be almost averaging on $U_i\subset {\mathbb {E}}$ with violation $\varepsilon _i\ge 0$ and averaging constant $\alpha _i>0$ $(i=1,2)$ and define the set

$$\begin{aligned} U := \{x\in U_2 \mid R_{T_2,\lambda }x \subset U_1\}. \end{aligned}$$

Then $T_{\lambda }$ is almost averaging on *U* with violation $\varepsilon = \varepsilon _1+\varepsilon _2+(1+\lambda )\varepsilon _1\varepsilon _2$ and averaging constant $\alpha = \frac{2\max \{\alpha _1,\alpha _2\}}{1+(1+\lambda )\max \{\alpha _1,\alpha _2\}}$.

### Proof

By the implication (i) $\Rightarrow$ (ii) of Lemma 1, the operators $R_{T_i,\lambda } = (1+\lambda )T_i - \lambda \,\mathrm{Id}$ are almost averaging on $U_i$ with violation $(1+\lambda )\varepsilon _i$ and averaging constant $(1+\lambda )\alpha _i$ $(i=1,2)$. Then thanks to [38, Proposition 2.4 (iii)], the operator $T:=R_{T_1,\lambda }R_{T_2,\lambda }$ is almost averaging on *U* with violation $(1+\lambda )\left( \varepsilon _1+\varepsilon _2+(1+\lambda )\varepsilon _1\varepsilon _2\right)$ and averaging constant $\frac{2(1+\lambda )\max \{\alpha _1,\alpha _2\}}{1+(1+\lambda )\max \{\alpha _1,\alpha _2\}}$. Note that $T_{\lambda }=(1+\lambda )T -\lambda \,\mathrm{Id}$ by Proposition 1. We have by the implication (ii) $\Rightarrow$ (i) of Lemma 1 that $T_{\lambda }$ is almost averaging on *U* with violation $\varepsilon =\varepsilon _1+\varepsilon _2+(1+\lambda )\varepsilon _1\varepsilon _2$ and averaging constant $\alpha =\frac{2\max \{\alpha _1,\alpha _2\}}{1+(1+\lambda )\max \{\alpha _1,\alpha _2\}}$ as claimed. $\square$

We next discuss convergence of $T_{\lambda }$ based on the abstract results established in [38]. Our agenda is to verify the assumptions of Theorem 1. To simplify the exposure in terms of presentation, we have chosen to state the results corresponding to $S={{\mathrm{\mathsf {Fix}\,}}}T_{\lambda }$ and $\varLambda ={\mathbb {E}}$ in Theorem 1. In the sequel, we will denote, for a nonnegative real $\rho$,

$$\begin{aligned} S_\rho :={{\mathrm{\mathsf {Fix}\,}}}T_{\lambda } + \rho \mathbb {B}. \end{aligned}$$

### Theorem 2

(Convergence of algorithm $T_{\lambda }$ with metric subregularity) Let $T_{\lambda }$ be defined at (7), $\delta >0$ and $\gamma \in (0,1)$. Suppose that for each $n\in \mathbb {N}$, the following conditions are satisfied.

(i) $T_2$ is almost averaging on $S_{\gamma ^n\delta }$ with violation $\varepsilon _{2,n}\ge 0$ and averaging constant $\alpha _{2,n}\in (0,1)$, and $T_1$ is almost averaging on the set $S_{\gamma ^n\delta } \cup R_{T_2,\lambda }\left( S_{\gamma ^n\delta }\right)$ with violation $\varepsilon _{1,n}\ge 0$ and averaging constant $\alpha _{1,n}\in (0,1)$.
(ii) The mapping $T_{\lambda }-\,\mathrm{Id}$ is metrically subregular on $D_n:=S_{\gamma ^n\delta }\setminus S_{\gamma ^{n+1}\delta }$ for 0 with gauge $\mu _n$ satisfying
  11 $$\begin{aligned} \inf _{x\in D_n} \frac{\mu _n\left( \text {dist}\,\left( x,{{\mathrm{\mathsf {Fix}\,}}}T_{\lambda }\right) \right) }{\text {dist}\,\left( x,{{\mathrm{\mathsf {Fix}\,}}}T_{\lambda }\right) } \ge \kappa _n > \sqrt{\frac{\alpha _n\varepsilon _n}{1-\alpha _n}}, \end{aligned}$$
  where $$\varepsilon _n :=\varepsilon _{1,n}+\varepsilon _{2,n} + (1+\lambda )\varepsilon _{1,n}\varepsilon _{2,n}$$ and $$\alpha _n :=\frac{2\max \{\alpha _{1,n},\alpha _{2,n}\}}{1+(1+\lambda )\max \{\alpha _{1,n},\alpha _{2,n}\}}$$.

Then all iterations $x_{k+1}\in T_{\lambda }x_k$ starting in $S_{\delta }$ satisfy

12 $$\begin{aligned} \text {dist}\,\left( x_k,{{\mathrm{\mathsf {Fix}\,}}}T_{\lambda }\right) \rightarrow 0 \end{aligned}$$

and

13 $$\begin{aligned} \text {dist}\,\left( x_{k+1}, {{\mathrm{\mathsf {Fix}\,}}}T_{\lambda }\right) \le c_n\, \text {dist}\,\left( x_k,{{\mathrm{\mathsf {Fix}\,}}}T_{\lambda }\right) \quad \forall x_k\in D_n, \end{aligned}$$

where $$c_n:=\sqrt{1+\varepsilon _n-\frac{(1-\alpha _n)\kappa _n^2}{\alpha _n}}<1$$.

In particular, if $\left( \frac{(1-\alpha _n)\kappa _n^2}{\alpha _n}-\varepsilon _n\right)$ is bounded from below by some $\tau >0$ for all *n* sufficiently large, then the convergence (12) is R-linear with rate at most $\sqrt{1-\tau }$.

### Proof

For each $n\in \mathbb {N}$, we verify the assumptions of Theorem 1 for ${\mathcal {O}}=S_{\gamma ^n\delta }$, ${\mathcal {V}}= S_{\gamma ^{n+1}\delta }$ and $D_n={\mathcal {O}}\setminus {\mathcal {V}}= S_{\gamma ^n\delta }\setminus S_{\gamma ^{n+1}\delta }$. Under assumption (i) of Theorem 2, Proposition 2 ensures that $T_{\lambda }$ is almost averaging on $S_{\gamma ^n\delta }$ with violation $\varepsilon _n$ and averaging constant $\alpha _n$. In other words, condition (a) of Theorem 1 is satisfied with $\varepsilon =\varepsilon _n$ and $\alpha =\alpha _n$. Assumption (ii) of Theorem 2 also fulfills condition (b) of Theorem 1 with $\kappa =\kappa _n$ in view of Remark 2. Theorem 1 then yields the conclusion of Theorem 2 after a straightforward care of the involving quantitative constants. $\square$

The first inequality in (11) essentially says that the gauge function $\mu _n$ can be bounded from below by a linear function on the reference interval.

### Remark 3

In Theorem 2, the fundamental goal of formulating assumption (i) on the set $S_{\gamma ^n\delta }$ and assumption (ii) on the set $D_n$ is that one can characterize sublinear convergence of an iteration on $S_{\delta }$ via linear progression of its iterates through each of the annular set $D_n$. This idea is based on the fact that for larger *n*, the almost averaging property of $T_{\lambda }$ on $S_{\gamma ^n\delta }$ is always improved but the metric subregularity on $D_n$ may get worse, however, if the corresponding quantitative constants still satisfy condition (11), then convergence is guaranteed. For an illustrative example, we refer the reader to [38, Example 2.4].

## Application to feasibility

We consider algorithm $T_{\lambda }$ for solving feasibility problem involving two closed sets $A,B\subset {\mathbb {E}}$,

14 $$\begin{aligned} x^+\in T_{\lambda }x&=P_A\left( (1+\lambda )P_Bx-\lambda x\right) -\lambda \left( P_Bx-x\right) \nonumber \\&=P_AR_{P_B,\lambda }(x)-\lambda \left( P_Bx-x\right) . \end{aligned}$$

Note that $T_{\lambda }$ with $\lambda =0$ and 1 corresponds to the alternating projections $P_AP_B$ and the DR method $\frac{1}{2}(R_{A}\circ R_{B}+\,\mathrm{Id})$, respectively.

It is worth recalling that feasibility problem for $m\ge 2$ sets can be reformulated as a feasibility problem for two constructed sets on the product space ${\mathbb {E}}^m$ with one of the later sets is a linear subspace, and the regularity properties in terms of both individual sets and collections of sets of the later sets are inherited from those of the former ones [3, 32].

When *A* is an affine set, then the projector $P_A$ is affine and $T_{\lambda }$ is a convex combination of the alternating projection and the DR methods since

$$\begin{aligned} T_{\lambda }x&= P_A\left( (1-\lambda )P_Bx+\lambda (2P_Bx-x)\right) -\lambda \left( P_Bx-x\right) \\&=(1-\lambda )P_AP_Bx + \lambda \left( x + P_A(2P_Bx-x) - P_Bx\right) \\&= (1-\lambda )T_0(x) + \lambda T_1(x). \end{aligned}$$

In this case, we establish convergence results for all convex combinations of the alternating projection and the DR methods. To our best awareness, this kind of results seems to be new.

Recall that when applied to inconsistent feasibility problems the DR operator has no fixed points. We next show that the set of fixed points of $T_{\lambda }$ with $\lambda \in [0,1)$ for convex inconsistent feasibility problems is nonempty. This result follows the lines of [36, Lemma 2.1] where the fixed point set of the RAAR operator is characterized.

### Proposition 3

(Fixed points of $T_{\lambda }$ for convex inconsistent feasibility) For closed convex sets $A,B\subset \mathbb {E}$, let $G=\overline{B-A}$, $g=P_G0$, $E=A\cap (B-g)$ and $F=(A+g)\cap B$. Then

$$\begin{aligned} {{\mathrm{\mathsf {Fix}\,}}}T_{\lambda }=E-\frac{\lambda }{1-\lambda }g\quad \forall \lambda \in [0,1). \end{aligned}$$

### Proof

We first show that $E-\frac{\lambda }{1-\lambda }g\subset {{\mathrm{\mathsf {Fix}\,}}}T_{\lambda }$. Pick any $e\in E$ and denote $f=e+g\in F$ as definitions of *E* and *F*. We are checking that

$$\begin{aligned} x:=e-\frac{\lambda }{1-\lambda }g\in {{\mathrm{\mathsf {Fix}\,}}}T_{\lambda }. \end{aligned}$$

Since $x=f-\frac{1}{1-\lambda }g$ and $-g\in N_B(f)$, we get $P_Bx=f$.

Analogously, since $g\in N_A(e)$ and

$$\begin{aligned} (1+\lambda )P_Bx-\lambda x=(1+\lambda )f-\lambda x=e+\frac{1}{1-\lambda }g, \end{aligned}$$

we have $$P_A((1+\lambda )P_Bx-\lambda x)=e$$.

Hence,

$$\begin{aligned} x-T_{\lambda }x=\;&x-P_A\left( (1+\lambda )P_Bx-\lambda x\right) +\lambda \left( P_Bx-x\right) \\ =\;&x-e+\lambda \left( f-x\right) =0. \end{aligned}$$

That is $$x\in {{\mathrm{\mathsf {Fix}\,}}}T_{\lambda }$$.

We next show that ${{\mathrm{\mathsf {Fix}\,}}}T_{\lambda }\subset E-\frac{\lambda }{1-\lambda }g$. Pick any $x\in {{\mathrm{\mathsf {Fix}\,}}}T_{\lambda }$. Let $f=P_Bx$ and $y=x-f$. Thanks to $x\in {{\mathrm{\mathsf {Fix}\,}}}T_{\lambda }$ and the definition of $T_{\lambda }$,

15 $$\begin{aligned} P_A((1+\lambda )P_Bx-\lambda x)=\;&\lambda (P_Bx-x)+x \nonumber \\ =\;&-\lambda y+y+f=f+(1-\lambda )y. \end{aligned}$$

Now, for any $a\in A$, since *A* is closed and convex, we have

$$\begin{aligned} 0\ge \;&\left\langle a-P_A((1+\lambda )P_Bx-\lambda x), (1+\lambda )P_Bx-\lambda x-P_A((1+\lambda )P_Bx-\lambda x) \right\rangle \\ =\;&\left\langle a-(f+(1-\lambda )y), (1+\lambda )f-\lambda x-(f+(1-\lambda )y) \right\rangle \\ =\;&\left\langle a-f-(1-\lambda )y, -y \right\rangle = \left\langle -a+f, y \right\rangle +(1-\lambda )\left\| y\right\| ^2. \end{aligned}$$

On the other hand, for any $b\in B$, since *B* is closed and convex, we have

$$\begin{aligned} \left\langle b-f, y \right\rangle = \left\langle b-f, x-f \right\rangle =\left\langle b-P_Bx, x-P_Bx \right\rangle \le 0. \end{aligned}$$

Combining the last two inequalities yields

$$\begin{aligned} \left\langle b-a, y \right\rangle \le -(1-\lambda )\left\| y\right\| ^2\le 0\quad \forall a\in A,\, \forall b\in B. \end{aligned}$$

Take a sequence $(a_n)$ in *A* and a sequence $(b_n)$ in *B* such that $g_n:=b_n-a_n\rightarrow g$. Then

16 $$\begin{aligned} \left\langle g_n, y \right\rangle \le -(1-\lambda )\left\| y\right\| ^2\le 0\quad \forall n. \end{aligned}$$

Taking the limit and using the Cauchy–Schwarz inequality yields

$$\begin{aligned} \left\| y\right\| \le \frac{1}{1-\lambda }\left\| g\right\| . \end{aligned}$$

Conversely, by (15) with noting that $f\in B$ and $P_A((1+\lambda )P_Bx-\lambda x)\in A$,

$$\begin{aligned} \left\| y\right\| =\frac{1}{1-\lambda }\left\| f-P_A((1+\lambda )P_Bx-\lambda x)\right\| \ge \frac{1}{1-\lambda }\left\| g\right\| . \end{aligned}$$

Hence $\left\| y\right\| =\frac{1}{1-\lambda }\left\| g\right\|$, and taking the limit in (16), which yields $y=-\frac{1}{1-\lambda }g$. Since $f\in B$ and $f-g=f+(1-\lambda )y=P_A((1+\lambda )P_Bx-\lambda x)\in A$, we have $f-g\in A\cap (B-g)=E$ and, therefore,

$$\begin{aligned} x=f+y=f-\frac{1}{1-\lambda }g=f-g-\frac{\lambda }{1-\lambda }g\in E-\frac{\lambda }{1-\lambda }g. \end{aligned}$$

$$\square $$

We next discuss the two key ingredients for convergence of algorithm $T_{\lambda }$ applied to feasibility problems: 1) almost averagedness of $T_{\lambda }$, and 2) metric subregularity of $T_{\lambda }-\,\mathrm{Id}$. The two properties will be deduced from the $(\varepsilon ,\delta )$-regularity of the individual sets and the transversality of the collection of sets, respectively.

The next proposition shows averagedness of $T_{\lambda }$ applied to feasibility problems involving $(\varepsilon ,\delta )$-regular sets.

### Proposition 4

Let *A* and *B* be $(\varepsilon ,\delta )$-regular at $\bar{x}\in A\cap B$ and define the set

17 $$\begin{aligned} U:=\{x\in {\mathbb {E}} \mid P_Bx\subset {\mathbb {B}}_{\delta }(\bar{x}) \text{ and } P_AR_{P_B,\lambda }x\subset {\mathbb {B}}_{\delta }(\bar{x})\}. \end{aligned}$$

Then $T_{\lambda }$ is pointwise almost averaging on *U* at every point $z \in S:=A\cap B\cap {\mathbb {B}}_{\delta }(\bar{x})$ with averaging constant $\frac{2}{3+\lambda }$ and violation

18 $$\begin{aligned} \tilde{\varepsilon }:=2(2\varepsilon +2\varepsilon ^2) + (1+\lambda )(2\varepsilon +2\varepsilon ^2)^2. \end{aligned}$$

### Proof

Let us define the two sets

$$\begin{aligned} U_A :=\{y\in {\mathbb {E}} \mid P_Ay\subset {\mathbb {B}}_{\delta }(\bar{x})\},\quad U_B :=\{x\in {\mathbb {E}} \mid P_Bx\subset {\mathbb {B}}_{\delta }(\bar{x})\} \end{aligned}$$

and note that $x\in U$ if and only if $x\in U_B$ and $R_{P_B,\lambda }x\subset U_A$. Thanks to Lemma 2 (iii), $R_{P_A,\lambda }$ and $R_{P_B,\lambda }$ are pointwise almost averaging at every point $z\in S$ with violation $(1+\lambda )(2\varepsilon +2\varepsilon ^2)$ and averaging constant $\frac{1+\lambda }{2}$ on $U_A$ and $U_B$, respectively. Then due to [38, Proposition 2.4 (iii)], the operator $T:=R_{P_A,\lambda }R_{P_B,\lambda }$ is pointwise almost averaging on *U* at every point $z\in S$ with averaging constant $\frac{2(1+\lambda )}{3+\lambda }$ and violation $(1+\lambda )\tilde{\varepsilon }$, where $\tilde{\varepsilon }$ is given by (18). Note that $T_{\lambda }=(1+\lambda )T -\lambda \,\mathrm{Id}$ by Proposition 1. Thanks to Lemma 1, $T_{\lambda }$ is pointwise almost averaging on *U* at every point $z \in S$ with violation $\tilde{\varepsilon }$ and averaging constant $\frac{2}{3+\lambda }$ as claimed. $\square$

### Remark 4

It follows from Lemma 2 (i) & (iii) that the set *U* defined by (17) contains at least the ball ${\mathbb {B}}_{\delta '}(\bar{x})$, where

$$\begin{aligned} \delta ' :=\frac{\delta }{2(1+\varepsilon )\sqrt{1+(1+\lambda )(2\varepsilon +2\varepsilon ^2)}}>0. \end{aligned}$$

We next integrate Proposition 4 into Theorem 2 to obtain convergence of algorithm $T_{\lambda }$ for solving consistent feasibility problems involving $(\varepsilon ,\delta )$-regular sets.

### Corollary 1

(Convergence of algorithm $T_{\lambda }$ for feasibility) Consider the algorithm $T_{\lambda }$ defined at (14) and suppose that ${{\mathrm{\mathsf {Fix}\,}}}T_{\lambda } = A\cap B \ne \emptyset$. Denote $S_\rho = {{\mathrm{\mathsf {Fix}\,}}}T_{\lambda } + \rho \mathbb {B}$ for a nonnegative real $\rho$. Suppose that there are $\delta >0$, $\varepsilon \ge 0$ and $\gamma \in (0,1)$ such that *A* and *B* are $(\varepsilon ,\delta ')$-regular at avery point $z\in A\cap B$, where

$$\begin{aligned} \delta ':=2\delta (1+\varepsilon )\sqrt{1+(1+\lambda )(2\varepsilon +2\varepsilon ^2)}, \end{aligned}$$

and for each $n\in \mathbb {N}$, the mapping $T_{\lambda }-\,\mathrm{Id}$ is metrically subregular on $D_n:=S_{\gamma ^n\delta }\setminus S_{\gamma ^{n+1}\delta }$ for 0 with gauge $\mu _n$ satisfying

$$\begin{aligned} \inf _{x\in D_n} \frac{\mu _n\left( \text {dist}\,\left( x,A\cap B\right) \right) }{\text {dist}\,\left( x,A\cap B\right) } \ge \kappa _n > \sqrt{\frac{2\tilde{\varepsilon }}{1+\lambda }}, \end{aligned}$$

where $\tilde{\varepsilon }$ is given at (18).

Then all iterations $x_{k+1}\in T_{\lambda }x_k$ starting in $S_{\delta }$ satisfy (12) and (13) with $c_n:=\sqrt{1+\tilde{\varepsilon } - \frac{(1+\lambda )\kappa _n^2}{2}}<1$.

In particular, if $(\kappa _n)$ is bounded from below by some ${\kappa }>\sqrt{\frac{2\tilde{\varepsilon }}{1+\lambda }}$ for all *n* sufficiently large, then $(x_k)$ eventually converges R-linearly to a point in $A\cap B$ with rate at most $\sqrt{1+\tilde{\varepsilon } - \frac{(1+\lambda ){\kappa }^2}{2}}<1$.

### Proof

Let any $x\in D_n$, for some $n\in \mathbb {N}$, $x^+ \in T_{\lambda }x$ and $\bar{x}\in P_{A\cap B}x$. A combination of Proposition 4 and Remark 4 implies that $T_{\lambda }$ is pointwise almost averaging on ${\mathbb {B}}_{\delta }(\bar{x})$ at every point $z\in A\cap B\cap {\mathbb {B}}_{\delta }(\bar{x})$ with violation $\tilde{\varepsilon }$ given by (18) and averaging constant $\frac{2}{3+\lambda }$. In other words, condition (a) of Theorem 1 is satisfied. Condition (b) of Theorem 1 is also fulfilled by the same argument as the one used in Theorem 2. The desired conclusion now follows from Theorem 1. $\square$

In practice, the metric subregularity assumption is often more challenging to be verified than the averaging property. In the concrete example of consistent alternating projections $P_AP_B$, that metric subregularity condition holds true if and only if the collection of sets is subtransversal. We next show that the metric subregularity of $T_{\lambda }-\,\mathrm{Id}$ can be deduced from the transversality of the collection of sets $\{A,B\}$. As a result, if the sets are also sufficiently regular, then local linear convergence of the iteration $x_{k+1}\in T_{\lambda }x_k$ is guaranteed.

We first describe the concept of relative transversality of collections of sets. In the sequel, we set $\varLambda :=\mathrm{aff}(A\cup B)$, the smallest affine set in ${\mathbb {E}}$ containing both *A* and *B*.

### Assumption 3

The collection $\{A,B\}$ is transversal at $\bar{x}\in A\cap B$ relative to $\varLambda$ with constant $\bar{\theta }<1$, that is, for any $\theta \in (\bar{\theta }, 1)$, there exists $\delta >0$ such that

$$\begin{aligned} \left\langle u, v \right\rangle \ge -\theta \left\| u\right\| \cdot \left\| v\right\| \end{aligned}$$

holds for all $a\in A\cap {\mathbb {B}}_{\delta }(\bar{x})$, $b\in B\cap {\mathbb {B}}_{\delta }(\bar{x})$, $u\in N_{A|\varLambda }^{\text {prox}}(a)$ and $v\in N_{B|\varLambda }^{\text {prox}}(b)$.

Thanks to [22, Theorem 1] and [28, Theorem 1], Assumption 3 also ensures subtransversality of $\{A,B\}$ at $\bar{x}$ relative to $\varLambda$ with constant at least $\sqrt{\frac{1-\theta }{2}}$ on the neighborhood ${\mathbb {B}}_{\delta }(\bar{x})$, that is

19 $$\begin{aligned} \sqrt{\frac{1-\theta }{2}}\, \text {dist}\,(x,A\cap B) \le \max \{\text {dist}\,(x,A),\text {dist}\,(x,B)\}\quad \forall x\in \varLambda \cap {\mathbb {B}}_{\delta }(\bar{x}). \end{aligned}$$

The next lemma is at the heart of our subsequent discussion.

### Lemma 3

Suppose that Assumption 3 is satisfied. Then for any $\theta \in (\bar{\theta },1)$, there exists a number $\delta >0$ such that for all $x\in {\mathbb {B}}_{\delta }(\bar{x})$ and $x^+ \in T_{\lambda }x$,

20 $$\begin{aligned} \kappa \, \text {dist}\,(x,{A\cap B}) \le \left\| x-x^+\right\| , \end{aligned}$$

where $\kappa$ is defined by

21 $$\begin{aligned} \kappa :=\frac{(1-\theta )\sqrt{1+\theta }}{\sqrt{2}\max \left\{ 1,\lambda +\sqrt{1-\theta ^2}\right\} }>0. \end{aligned}$$

### Proof

For any $\theta \in (\bar{\theta },1)$, there is a number $\delta >0$ satisfying the property described in Assumption 3. Let us set $\delta ' = \delta /6$ and show that condition (20) is fulfilled with $\delta '$.

Indeed, let us consider any $x\in {\mathbb {B}}_{\delta '}(\bar{x})$, $b\in P_Bx$, $y=(1+\lambda )b-\lambda x$, $a\in P_Ay$ and $x^+=a-\lambda (b-x)\in T_{\lambda }x$. From the choice of $\delta '$, it is clear that $a,b\in {\mathbb {B}}_{\delta }(\bar{x})$. Since $x-b\in N_{B|\varLambda }^{\text {prox}}(b)$ and $y-a\in N_{A|\varLambda }^{\text {prox}}(a)$, Assumption 3 yields that

22 $$\begin{aligned} \left\langle x-b, y-a \right\rangle \ge -\theta \left\| x-b\right\| \cdot \left\| y-a\right\| . \end{aligned}$$

By the definition of $T_{\lambda }$, we have

23 $$\begin{aligned} \left\| x-x^+\right\| ^2&= \left\| x-b+y-a\right\| ^2 \nonumber \\&= \left\| x-b\right\| ^2+\left\| y-a\right\| ^2+2\left\langle x-b, y-a \right\rangle \nonumber \\&\ge \left\| x-b\right\| ^2+\left\| y-a\right\| ^2-2\theta \left\| x-b\right\| \cdot \left\| y-a\right\| \nonumber \\&\ge \left( 1-\theta ^2\right) \left\| x-b\right\| ^2 = \left( 1-\theta ^2\right) \, \text {dist}\,^2(x,B), \end{aligned}$$

where the first inequality follows from (22).

We will take care of the two possible cases regarding $\text {dist}\,(x,A)$ as follows.

*Case 1* $\text {dist}\,(x,A)\le \left( \lambda +\sqrt{1-\theta ^2}\right) \, \text {dist}\,(x,B)$. Thanks to (23) we get

24 $$\begin{aligned} \left\| x-x^+\right\| ^2 \ge \frac{1-\theta ^2}{\left( \lambda +\sqrt{1-\theta ^2}\right) ^2}\,\text {dist}\,^2(x,A). \end{aligned}$$

*Case 2* $\text {dist}\,(x,A)> \left( \lambda +\sqrt{1-\theta ^2}\right) \,\text {dist}\,(x,B)$. By the triangle inequality and the construction of $T_{\lambda }$, we get

25 $$\begin{aligned} \left\| x-x^+\right\|&\ge \left\| x-a\right\| - \left\| a-x^+\right\| = \left\| x-a\right\| -\lambda \left\| x-b\right\| \nonumber \\&\ge \text {dist}\,(x,A) - \lambda \,\text {dist}\,(x,B) \ge \left( 1-\frac{\lambda }{\lambda +\sqrt{1-\theta ^2}}\right) \,\text {dist}\,(x,A). \end{aligned}$$

Since

$$\begin{aligned} \frac{1-\theta ^2}{\left( \lambda +\sqrt{1-\theta ^2}\right) ^2} = \left( 1-\frac{\lambda }{\lambda +\sqrt{1-\theta ^2}}\right) ^2, \end{aligned}$$

we always have from (24) and (25) that

26 $$\begin{aligned} \left\| x-x^+\right\| ^2 \ge \frac{1-\theta ^2}{\left( \lambda +\sqrt{1-\theta ^2}\right) ^2}\,\text {dist}\,^2(x,A). \end{aligned}$$

Combining (23), (26) and (19), we obtain

$$\begin{aligned} \left\| x-x^+\right\| ^2&\ge \frac{1-\theta ^2}{\max \left\{ {1,\left( \lambda +\sqrt{1-\theta ^2}\right) ^2}\right\} }\max \left\{ \text {dist}\,^2(x,A),\text {dist}\,^2(x,B)\right\} \\&\ge \frac{(1-\theta ^2)(1-\theta )}{2\max \left\{ {1,\left( \lambda +\sqrt{1-\theta ^2}\right) ^2}\right\} }\,\text {dist}\,^2(x,A \cap B), \end{aligned}$$

which yields (20) as claimed. $\square$

In the special case that $\lambda =1$, Lemma 3 refines [13, Lemma 3.14] and [45, Lemma 4.2] where the result was proved for the DR operator with an additional assumption on regularity of the sets.

The next result is the final preparation for our linear convergence result.

### Lemma 4

[45, Proposition 2.11] Let $T:{\mathbb {E}}\rightrightarrows {\mathbb {E}}$, $S\subset {\mathbb {E}}$ be closed and $\bar{x}\in S$. Suppose that there are $\delta >0$ and $c\in [0,1)$ such that for all $x\in {\mathbb {B}}_{\delta }(\bar{x})$, $x^+\in Tx$ and $z\in P_Sx$,

27 $$\begin{aligned} \left\| x^+-z\right\| \le c\left\| x-z\right\| . \end{aligned}$$

Then every iteration $x_{k+1}\in Tx_k$ starting sufficiently close to $\bar{x}$ converges R-linearly to a point $\tilde{x}\in S\cap {\mathbb {B}}_{\delta }(\bar{x})$. In particular,

$$\begin{aligned} \left\| x_k-\tilde{x}\right\| \le \frac{\left\| x_0-\bar{x}\right\| (1+c)}{1-c}\,c^k. \end{aligned}$$

We are now ready to prove local linear convergence for algorithm $T_{\lambda }$ which generalizes the corresponding results established in [13, 45] for the DR method.

### Theorem 4

(Linear convergence of algorithm $T_{\lambda }$ for feasibility) In addition to Assumption 3, suppose that *A* and *B* are $(\varepsilon ,\delta )$-regular at $\bar{x}$ with $\tilde{\varepsilon } < \frac{(1+\lambda )\kappa ^2}{2}$, where $\tilde{\varepsilon }$ and $\kappa$ are given by (18) and (21), respectively. Then every iteration $x_{k+1} \in T_{\lambda }x_k$ starting sufficiently close to $\bar{x}$ converges R-linearly to a point in $A\cap B$.

### Proof

Assumption 3 ensures the existence of $\delta _1>0$ such that Lemma 3 holds true. In view of Proposition 4 and Remark 4, one can find a number $\delta _2>0$ such that $T_{\lambda }$ is pointwise almost averaging on ${\mathbb {B}}_{\delta _2}(\bar{x})$ at every point $z\in A\cap B\cap {\mathbb {B}}_{\delta _2}(\bar{x})$ with violation $\tilde{\varepsilon }$ given by (18) and averaging constant $\frac{2}{3+\lambda }$. Define $\delta '=\min \{\delta _1,\delta _2\}>0$.

Now let us consider any $x\in {\mathbb {B}}_{\delta '/2}(\bar{x})$, $x^+\in T_{\lambda }x$ and $z\in P_{A\cap B}x$. It is clear that $z\in {\mathbb {B}}_{\delta '}(\bar{x})$. Proposition 4 and Lemma 3 then respectively yield

28 $$\begin{aligned} \left\| x^+-z\right\| ^2&\le (1+\tilde{\varepsilon })\left\| x-z\right\| ^2 - \frac{1+\lambda }{2}\left\| x-x^+\right\| ^2, \end{aligned}$$

29 $$\begin{aligned} \left\| x-x^+\right\| ^2&\ge \kappa ^2\,\text {dist}\,^2(x,{A\cap B}) = \kappa ^2\left\| x-z\right\| ^2, \end{aligned}$$

where $\kappa$ is given by (21).

Substituting (29) into (28), we get

$$\begin{aligned} \left\| x^+-z\right\| ^2&\le \left( 1+\tilde{\varepsilon }-\frac{(1+\lambda )\kappa ^2}{2}\right) \left\| x-z\right\| ^2, \end{aligned}$$

which yields condition (27) of Lemma 4 and the desired conclusion now follows from this lemma. $\square$

## Application to sparse optimization

Our goal in this section is twofold: 1) to illustrate the linear convergence of algorithm $T_{\lambda }$ formulated in Theorem 4 via the *sparse optimization problem*, and 2) to demonstrate a promising performance of the algorithm $T_{\lambda }$ in comparison with the RAAR algorithm for this applied problem.

### Sparse optimization

We consider the *sparse optimization problem*

30 $$\begin{aligned} \min _{x\in {\mathbb {R}}^n} \left\| x\right\| _0 \quad \text{ subject } \text{ to } \quad Mx = b, \end{aligned}$$

where $M\in {\mathbb {R}}^{m\times n}$ $(m<n)$ is a full rank matrix, *b* is a given vector in ${\mathbb {R}}^m$, and $\left\| x\right\| _0$ is the number of nonzero entries of the vector *x*. The sparse optimization problem with complex variable is defined analogously by replacing ${\mathbb {R}}$ by ${\mathbb {C}}$ everywhere in the above model.

Many strategies for solving (30) have been proposed. We refer the reader to the famous paper by Candès and Tao [9] for solving this problem by using convex relaxations. On the other hand, assuming to have a good guess on the sparsity of the solutions to (30), one can tackle this problem by solving the *sparse feasibility problem* [14] of finding

31 $$\begin{aligned} \bar{x} \in A_s \cap B, \end{aligned}$$

where $$A_s :=\{x\in {\mathbb {R}}^n\mid \left\| x\right\| _0\le s\}$$ and $$B :=\{x\in {\mathbb {R}}^n\mid Mx = b\}$$.

It is worth mentioning that the initial guess *s* of the true sparsity is not numerically sensitive with respect to various projection methods, that is, for a relatively wide range of values of *s* above the true sparsity, projection algorithms perform very much in the same nature. Note also that the approach via sparse feasibility does not require convex relaxations of (30) and thus can avoid the likely expensive increase of dimensionality.

We run the two algorithms $T_{\lambda }$ and RAAR to solve (31) and compare their numerical performances. By taking *s* smaller than the true sparsity, we can also compare their performances for inconsistent feasibility.

Since *B* is affine, there is the closed algebraic form for the projector $P_B$,

$$\begin{aligned} P_Bx = x - M^{\dagger }(Mx-b)\quad \forall x\in {\mathbb {R}}^n, \end{aligned}$$

where $M^{\dagger }:= M^T(MM^T)^{-1}$ is the *Moore–Penrose inverse* of *M*. We have denoted $M^T$ the transpose matrix of *M* and taken into account that *M* is full rank. There is also a closed form for $P_{A_s}$ [6]. For each $x\in {\mathbb {R}}^n$, let us denote ${\mathcal {I}}_s(x)$ the set of all *s*-tubles of indices of *s* largest in absolute value entries of *x*. The set ${\mathcal {I}}_s(x)$ can contain multiple such *s*-tubles. The projector $P_{A_s}$ can be described as

$$\begin{aligned} P_{A_s}x = \left\{ z\in {\mathbb {R}}^n \mid \exists \; {\mathcal {I}}\in {\mathcal {I}}_s(x)\; \text{ such } \text{ that } \; z(k)= {\left\{ \begin{array}{ll} x(k) &{} \text{ if } k \in {\mathcal {I}}, \\ 0 &{} \text{ else } \end{array}\right. } \right\} . \end{aligned}$$

For convenience, we recall the two algorithms in this specific setting

$$\begin{aligned} RAAR_\beta&= \beta \left( P_{A_s}(2P_B-\,\mathrm{Id})\right) + (1-2\beta )P_B + \beta \,\mathrm{Id}, \\ T_{\lambda }&= P_{A_s}\left( (1+\lambda )P_B-\lambda \,\mathrm{Id}\right) - \lambda (P_B-\,\mathrm{Id}). \end{aligned}$$

### Convergence analysis

We analyze the convergence of algorithm $T_{\lambda }$ for the sparse feasibility problem (31). The next theorem establishes local linear convergence of algorithm $T_{\lambda }$ for solving sparse feasibility problems.

#### Theorem 5

(Linear convergence of algorithm $T_{\lambda }$ for sparse feasibility) Let $\bar{x} = (\bar{x}_i) \in A_s \cap B$ and suppose that *s* is the sparsity of the solutions to the problem (30). Then any iteration $x_{k+1}\in T_{\lambda }x_k$ starting sufficiently close to $\bar{x}$ converges R-linearly to $\bar{x}$.

#### Proof

We first show that $\bar{x}$ is an isolated point of $A_s \cap B$. Since *s* is the sparsity of the solutions to (30), we have that $\left\| \bar{x}\right\| _0=s$ and the set ${\mathcal {I}}_s(\bar{x})$ contains a unique element, denoted ${\mathcal {I}}_{\bar{x}}$. Note that $E_{\bar{x}}:= \mathrm{span}\{e_i:i\in {\mathcal {I}}_{\bar{x}}\}$ is the unique *s*-dimensional space component of $A_s$ containing $\bar{x}$, where $\{e_i:1\le i\le n\}$ is the canonical basic of ${\mathbb {R}}^n$. Let us denote

$$\begin{aligned} \delta :=\min _{i\in {\mathcal {I}}_{\bar{x}}}\, |\bar{x}_i| >0. \end{aligned}$$

We claim that

32 $$\begin{aligned} A_s\cap {\mathbb {B}}_{\delta }(\bar{x})&= E_{\bar{x}} \cap {\mathbb {B}}_{\delta }(\bar{x}), \end{aligned}$$

33 $$\begin{aligned} E_{\bar{x}}\cap B&= \{\bar{x}\}. \end{aligned}$$

Indeed, for any $x = (x_i) \in A_s\cap {\mathbb {B}}_{\delta }(\bar{x})$, we have by definition of $\delta$ that $x_i \ne 0$ for all $i\in {\mathcal {I}}_{\bar{x}}$. Hence $\Vert x\Vert _0=s$ and $x\in E_{\bar{x}}\cap {\mathbb {B}}_{\delta }(\bar{x})$. This proves (32).

For (33), it suffices to show the singleton of $E_{\bar{x}}\cap B$ since we already know that $\bar{x}\in E_{\bar{x}}\cap B$. Suppose otherwise that there exists $x = (x_i) \in E_{\bar{x}}\cap B$ with $x_j\ne \bar{x}_j$ for some index *j*. Since both $E_{\bar{x}}$ and *B* are affine, the intersection $E_{\bar{x}}\cap B$ contains the line $\{x + t(\bar{x}-x): t\in {\mathbb {R}}\}$ passing *x* and $\bar{x}$. In particular, it contains the point $z:= x + \frac{x_j}{x_j-\bar{x}_j}(\bar{x}-x)$. Then we have that $z\in B$ and $\Vert z\Vert _0\le s-1$ as $z_j=0$. This contradicts to the assumption that *s* is the sparsity of the solutions to (30), and hence (33) is proved.

A combination of (32) and (33) then yields

34 $$\begin{aligned} A_s \cap B \cap {\mathbb {B}}_{\delta }(\bar{x}) = E_{\bar{x}} \cap B \cap {\mathbb {B}}_{\delta }(\bar{x}) = \{\bar{x}\}. \end{aligned}$$

This means that $\bar{x}$ is an isolated point of $A_s\cap B$ as claimed. Moreover, the equalities in (34) imply that

$$\begin{aligned} P_{A_s}x = P_{E_{\bar{x}}}x\quad \forall x\in {\mathbb {B}}_{\delta /2}(\bar{x}). \end{aligned}$$

Therefore, for any starting point $x_0\in {\mathbb {B}}_{\delta /2}(\bar{x})$, the iteration $x_{k+1}\in T_{\lambda }x_k$ for solving (31) is identical to that for solving the feasibility problem for the two sets $E_{\bar{x}}$ and *B*. Since $E_{\bar{x}}$ and *B* are two affine subspaces intersecting at the unique point $\bar{x}$ by (33), the collection of sets $\{E_{\bar{x}},B\}$ is transversal at $\bar{x}$ relative to the affine hull $\mathrm{aff}(E_{\bar{x}}\cup B)$. Theorem 4 now can be applied to conclude that the iteration $x_{k+1}\in T_{\lambda }x_k$ converges R-linearly to $\bar{x}$. The proof is complete. $\square$

It is worth mentioning that the convergence analysis in Theorem 5 is also valid for the RAAR algorithm.

### Numerical experiment

We now set up a toy example as in [9, 14] which involves an unknown true object $\bar{x} \in {\mathbb {R}}^{256^2}$ with $\left\| \bar{x}\right\| _0=328$ (the sparsity rate is .005). Let *b* be 1 / 8 of the measurements of $F(\bar{x})$, the *Fourier transform* of $\bar{x}$, with the *sample indices* denoted ${\mathcal {J}}$. The *Poisson noise* was added when calculating the measurement *b*. Note that since $\bar{x}$ is real, $F(\bar{x})$ is *conjugate symmetric*, we indeed have nearly a double number of measurements. In this setting, we have

$$\begin{aligned} B=\{x\in {\mathbb {C}}^{256^2} \mid F(x)(k) = b(k), \; \forall k\in {\mathcal {J}}\}, \end{aligned}$$

and the two prox operators, respectively, take the forms

$$\begin{aligned} P_{A_s}x&= \left\{ z\in {\mathbb {R}}^n \mid \exists \; {\mathcal {I}}\in {\mathcal {I}}_s(x)\; \text{ such } \text{ that } \; z(k)= {\left\{ \begin{array}{ll} \mathrm{Re}\left( x(k)\right) &{} \text{ if } k \in {\mathcal {I}}, \\ 0 &{} \text{ else } \end{array}\right. } \right\} , \\ P_Bx&= F^{-1}(\hat{x}),\; \text{ where } \; \hat{x}(k) = {\left\{ \begin{array}{ll} b(k) &{} \text{ if } k \in {\mathcal {J}}, \\ F(x)(k) &{} \text{ else, } \end{array}\right. } \end{aligned}$$

where $\mathrm{Re}(x(k))$ denotes the real part of the complex number *x*(*k*), and $F^{-1}$ is the *inverse Fourier transform*.

The initial point was chosen randomly, and a warm-up procedure with 10 DR iterates was performed before running the two algorithms. The stopping criterion $\left\| x-x^+\right\| < 10^{-10}$ was used. We have used the *Matlab ProxToolbox* [37] to run this numerical experiment. The parameters were chosen in such a way that the performance is seemingly optimal for both algorithms. We chose $\beta =.65$ for the RAAR algorithm and $\lambda =.45$ for algorithm $T_{\lambda }$ in the case of consistent feasibility problem corresponding to $s=340$, and $\beta =.6$ for the RAAR algorithm and $\lambda =.4$ for algorithm $T_{\lambda }$ in the case of inconsistent feasibility problem corresponding to $s=310$.

The *change* of distances between two consecutive iterates is of interest. When linear convergence appears to be the case, it can yield useful information of the convergence rate. Under the assumption that the iterates will remain in the convergence area, one can obtain error bounds for the distance from the current iterate to a nearest solution. We also pay attention to the *gaps* in iterates that in a sense measure the infeasibility at the iterates. If we think feasibility problem as the problem of minimizing the sum of the squares of the distance functions to the sets, then gaps in iterates are the values of that function evaluated at the iterates. For the two algorithms under consideration, the iterates are themselves not informative but their shadows, by which we mean the projections of the iterates on one of the sets. Hence, the gaps in iterates are calculated for the iterate shadows instead of the iterates themselves.

**Fig. 1:**
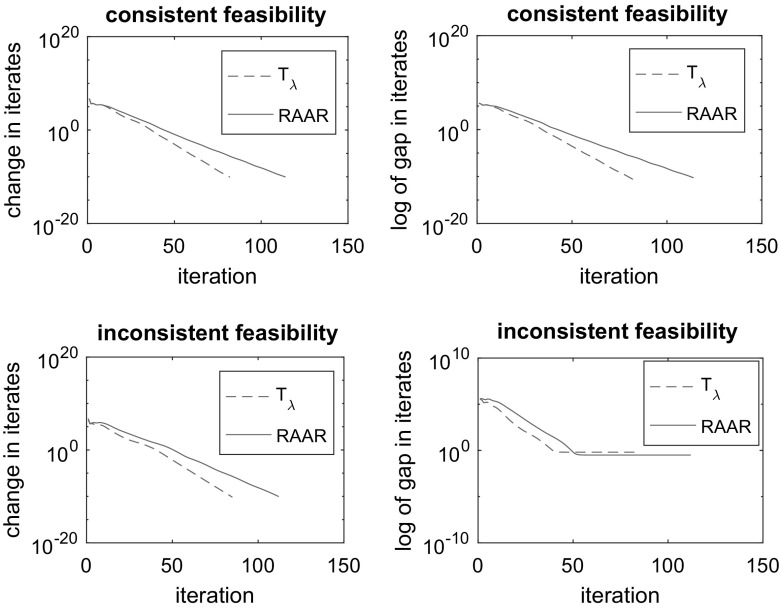
Performances of the RAAR and $T_{\lambda }$ algorithms for sparse feasibility problem: iterate changes in consistent case (top-left), iterate gaps in consistent case (top-right), iterate changes in inconsistent case (bottom-left) and iterate gaps in inconsistent case (bottom-right)

Figure 1 summarizes the performances of the two algorithms for both consistent and inconsistent sparse feasibility problems. We first emphasize that the algorithms appear to be convergent in both cases of feasibility. For the consistent case, algorithm $T_{\lambda }$ appears to perform better than the RAAR algorithm in terms of both the iterate changes and gaps. Also, the CPU time of algorithm $T_{\lambda }$ is around $10\%$ less than that of the RAAR algorithm. For the inconsistent case, we have a similar observation except that the iterate gaps for the RAAR algorithm are slightly better (smaller) than those for algorithm $T_{\lambda }$. Extensive numerical experiments in imaging problems illustrating the empirical performance of algorithm $T_{\lambda }$ will be the future work.
